# Finding Factors in Footfalls: Exploring the Factor Structure of Gait in Older Adults.

**DOI:** 10.1093/geroni/igab046.2475

**Published:** 2021-12-17

**Authors:** Michael Willden, Josh Palmer, Kristina Kowalski, Sandra Hundza, Stuart MacDonald

**Affiliations:** 1 University of Victoria, Victoria, British Columbia, Canada; 2 University of Victoria, University of Victoria, British Columbia, Canada; 3 University of Calgary, University of Calgary, Alberta, Canada

## Abstract

Gait is a reputed marker of global health spanning various bodily systems (MacDonald et al., 2017) and is a robust predictor of deleterious age-related outcomes (Van Kan et al., 2009). However, the sheer number of individual gait variables employed as predictors in the existing literature can obscure interpretations. To address this issue, researchers have explored the factor structure of gait indicators to explain variance in age-related gait performance, identifying disparate models characterized by three to five underlying latent gait constructs comprised of 8 to 23 indicators (Hollman et al., 2011; Lord et al., 2013). Beyond this heterogeneity, additional limitations characterizing this literature include solutions that assume statistical independence among gait constructs, as well as inclusion of severely multicollinear indicators. Using data from the Healthy Minds Healthy Bodies (HMHB) study, the present research focused upon replicating and contrasting previous factor analytic efforts. HMHB participants (n=128) were healthy community-dwelling adults (Mage=72.81±5.24 years; female=100). Gait indicators from a GAITRite computerized walkway were selected according to a priori theoretical rationale, compatibility with previous studies, and consideration of multicollinearity. Gait factor structure was initially analyzed using principal component analysis. Results indicate the presence of three latent gait domains reflecting pace, rhythm, and variability, accounting for over 82.4% of the variance in gait performance. Current proceedings involve implementing confirmatory factor analysis to compare competing gait models. Findings will address disparities across factor models in the gait literature, as well as discuss the optimal number of factors for describing the underlying dimensionality of gait.

